# Impact of Dobbs on Evaluation and Treatment of Ectopic Pregnancy: National Survey of Emergency Physicians

**DOI:** 10.5811/westjem.41205

**Published:** 2025-07-13

**Authors:** Monica Saxena, Dara Kass, Esther Choo

**Affiliations:** * Stanford University School of Medicine, Department of Emergency Medicine, Stanford, California; † Saint Francis Hospital, Department of Emergency Medicine, New York, New York; ‡ Oregon Health and Science University, Department of Emergency Medicine, Portland, Oregon

## Abstract

**Introduction:**

Inconsistent and ever-changing state abortion laws across the United States raise the possibility of deviation from established standards of emergency care. Yet the experiences of emergency physicians in this era have not been captured. We sought to examine the experiences of US emergency physicians in the management of presumed ectopic pregnancy since the *Dobbs* Supreme Court ruling and passage of new abortion restrictions affecting clinical decision-making around pregnancy termination.

**Methods:**

This was a cross-sectional survey of US emergency physicians administered online between April 1–15, 2024. The survey was completed by 150 board-certified US emergency physicians—50 physicians each from states categorized as abortion restrictive, semi-restrictive, or permissive—who were queried about any reported delays in or adaptations to the assessment and/or management of patients with known or suspected ectopic pregnancy.

**Results:**

We found that 24% of physicians in restrictive or semi-restrictive states reported delays in the management of patients with suspected or confirmed ectopic pregnancy, and 54% of physicians reported adaptations to care of these patients including repeat testing and arranging alternative care in cases where they might previously have delivered definitive care in the emergency department.

**Conclusion:**

In a post-*Dobbs* practice environment, emergency physicians across the United States, practicing in states with various abortion restrictions, reported delays and adaptations of care for patients with presumed or suspected ectopic pregnancy including deviations from standard of care in emergency medicine.

## BACKGROUND

In June 2022, the US Supreme Court ruling, *Dobbs v Jackson Women’s Health Organization*, reversed the constitutional protection of abortion and returned the power to regulate abortion access to individual states. In the wake of this decision, many states placed new restrictions on abortion.^1^ As of this writing, 21 states have restrictions on abortion access that would not have been possible prior to the passage of *Dobbs*.^2^ The practical implication of these laws is that healthcare clinicians may be unsure whether they are violating laws when they provide any care that includes pregnancy termination, including the treatment of ectopic pregnancy.

Management of ectopic pregnancy, a time-sensitive, often lethal condition, is a stark use case for the impact of legal restrictions on emergency care, as its unambiguous treatment is a medical or surgical abortion. Ruptured ectopic pregnancy is the leading cause of first trimester maternal death.^3^ For over a century, pregnancy termination has been established as the standard of care for ectopic pregnancy, with medical termination being used for over 50 years.^34^ Since the inception of emergency medicine as a specialty there has been no change in the best practices for treatment of ectopic pregnancy.^4^,^5^ In the best of circumstances, effective, life-saving management for ectopic pregnancy is challenging due to the initial silent nature of the condition and how rapidly individuals may progress to clinical instability. Delays in care or lack of adherence to standard clinical assessment and management place patients at risk for poor maternal outcomes. Even though the American College of Obstetricians and Gynecologists (ACOG) distinguishes an elective abortion from a medically necessary abortion (such as in the case of ectopic pregnancy) physicians including obstetricians and emergency physicians have been confronted with institutional and clinical barriers when treating patients with pregnancy termination for ectopic pregnancy.

The experiences of obstetricians practicing under restrictive abortion policies have been described previously.^6^ The objective of this study is to explore the experiences of U.S. emergency physicians in the management of ectopic pregnancy since Dobbs and the subsequent implementation of restrictive state abortion laws.

## METHODS

This was a cross-sectional survey of a convenience sample of clinically active US emergency physicians administered online over a two-week period between April–15, 2024. The questionnaire addressed changes in clinical care during the period of interest, defined as the time since the *Dobbs* decision in June 2022. We developed the initial questionnaire, which related to a variety of aspects of emergency reproductive healthcare, and then refined it through iterative rounds of expert review and face validity testing with practicing emergency physicians. For this study, we evaluated questions specifically related to the assessment and management of ectopic pregnancy (see Supplement A), which consisted of 8–11 items, depending on skip logic.

We divided states into three general categories, based on the policies in place at the time of survey dissemination, in March 2024: restrictive; semi-restrictive; and permissive states.^7^–^9^ We defined “restrictive” states as those with bans from conception to six weeks, “semi-restrictive” states as those with new bans implemented since the *Dobbs* decision but that still allowed for post-six-week abortion access, and “permissive” states as those that either had no new restrictions or had expanded protections for abortion access since June 2022. The states in each category are shown in Supplement B.

Those working in restrictive and semi-restrictive states were asked about delays in or adaptations to care of suspected or confirmed ectopic pregnancy. Permissive states were asked about calls or transfers into their state from more restrictive states. All respondents received questions about whether any new protocols or care plans were developed by their hospitals related to ectopic pregnancy. To ensure a common clinical understanding of care standards, we also included two clinical case questions (see questions 10 and 11 in Appendix) on management of ectopic pregnancy or potential ectopic pregnancy, using scenarios with an established standard of care.^10^

**Population Health Research Capsule**

What do we already know about this issue?

*The Supreme Court Dobbs decision has impacted emergency medicine (EM) clinical care of pregnant patients*.

What was the research question?

*How has EM physician clinical decision making changed post Dobbs with regards to the treatment of ectopic pregnancy?*

What was the major finding of the study?

*Twenty-four percent of EM physicians in abortion restrictive states reported delays or adaptations in care of patients with ectopic pregnancy*.

How does this improve population health?

*This study reveals that there are clinical impacts in emergency medicine with regards to care of patients with ectopic pregnancy following the Dobbs decision*.

The survey was distributed electronically by InCrowd,^10^ a company that administers health professional surveys.^11^ InCrowd validates physician status by National Provider Identifier and maintains current demographic information of physicians in its database, including age, sex, race and ethnicity, location of practice, and years since completion of residency training. The survey was closed to further participation within each category once the goal number of participants was achieved. Survey respondents received honoraria between $23–$31, depending on time spent completing the survey.

We calculated summary statistics (counts, means, percentages) for item responses by state categories. The study was approved by the Institutional Review Board of Stanford University.

## RESULTS

A total of 150 physicians, 50 respondents from each category, responded from 38 states. Participant characteristics are summarized in Table 1.

Twenty-four percent of respondents in restrictive and semi-restrictive states reported experiencing delays in care for patients with known or suspected ectopic pregnancy since the *Dobbs* decision (Table 2). Among those reporting delays, the most common reason for delays (58%) was needing a higher threshold of certainty for a definitive ectopic pregnancy diagnosis. Fifty-four percent of respondents in restrictive and semi-restrictive states reported adaptations in care made since the *Dobbs* decision (Table 3), including arranging close follow-up in cases where they might previously have delivered definitive care (31%) or obtaining additional imaging (26%) or beta-human chorionic gonadotropin (B-hcg) levels (24%) prior to treatment. Twenty percent of respondents in permissive states reported an increase in patients from abortion-restricted states coming to their ED for pregnancy-related care. Ten percent of respondents in these states reported receiving calls from clinicians regarding patients coming to their facility to receive pregnancy care due to restrictions in their states. Across all state categories, few physicians (7%) reported new protocols or care plans for patients with known or suspected ectopic pregnancy. Most emergency physicians responded to clinical scenarios in a manner consistent with clinical standards, without differences across state categories (75% restrictive states and 76% permissive states).

## DISCUSSION

Our results suggest that physicians are shifting practice in a way that has the potential to increase harmful outcomes among patients with ectopic pregnancies. A significant percentage of emergency physicians in states with post-*Dobbs* abortion restrictions–24%–reported delays in or adaptations to their management of patients with known or suspected ectopic pregnancy. Respondents reported requiring additional testing beyond a B-hcg level of 4000 milligrams per deciliter (mg/dL); this is above the current recommendation by ACOG for discriminatory zone of a B-hcg of 3500 mg/dL for decision-making in ectopic pregnancy.^12^

Such patients could be discharged from emergency departments (ED) with presumed or suspected ectopic pregnancies when they previously would have been treated with pregnancy termination or experienced delays in definitive care of their ectopic pregnancy with pregnancy termination.

The possibility of alterations in clinical practice for patients requiring pregnancy termination in EDs was anticipated by leaders in the field. Last year, the American College of Emergency Physicians, the largest national organization representing emergency practice, issued a policy statement, asserting that abortion “is a medical procedure, and as such [is a decision] to be made only by healthcare professionals with their patients.”^13^ This study’s data suggests concerns about impact on clinical decision-making were valid.

The role of EDs in pregnancy termination has come into focus due to questions about the authority of a 1986 federal law, the Emergency Medical Treatment & Labor Act (EMTALA), which defines a minimum standard for EDs, requiring that they ensure stability of those who present to care. Recently, in two cases, *Moyle v United States* and *Idaho v United States*,^14^ the Supreme Court was asked to determine the extent to which emergency physicians must balance obligations under EMTALA against state laws that have outlawed abortion care, even when provided for medical stabilization. In June, the Court dismissed the cases, sending them back to proceed through lower courts. The Supreme Court’s failure to rule decisively on EMTALA means that physicians will continue to face uncertainty about protections of care for patients requiring termination of a pregnancy as part of emergency care.

## LIMITATIONS

This study has several limitations. As an exploratory study, it was a non-random sample limited to 38 of the 50 states surveyed. Only physicians were surveyed, missing the input of physician assistants and nurse practitioners working in EDs. Participation bias may mean that those who completed the survey had relatively strong feelings about abortion bans, whether positive or negative, and may not reflect the general experience of practicing emergency physicians around the country. Recall bias may mean clinicians under- or over-reported cases occurring over the previous 22 months.

## CONCLUSION

This study reports, for the first time, perceived impacts on post-*Dobbs* restrictions on the emergency management of patients with suspected ectopic pregnancy. In states with new abortion restrictions 24% of the clinicians surveyed reported changes in clinical decision-making for what is commonly held to be a clearly defined exception to abortion bans, raising the possibility that other aspects of patient care previously assumed to be protected by clinical standards of care and good faith physician judgment are affected by these restrictions. Further research is needed to determine the impact of these restrictions on all aspects of reproductive health care in emergency medicine.

## Supplementary Information



## Figures and Tables

**Table 1:** Survey participant characteristics (N=150).

Years since residency graduation [mean (median)]	13 (12)
Sex [n (%)]	
Female	63 (42%)
Male	86 (57%)
Non-binary	1 (1%)
Race [n (%)]	
Asian	22 (15%)
Black	3 (2%)
White	115 (80%)
Other	3 (2%)
Hispanic/Latino [n (%)]	5 (3%)
Facility location [n (%)]	
Critical access	4 (3%)
Rural	17 (11%)
Suburban	71 (47%)
Urban	58 (39%)
Facility type* [n (%)]	
Academic	10 (15%)
Academic affiliated	28 (19%)
Community	70 (47%)
Freestanding	21 (14%)
Other**	16 (11%)

* Due to rounding, total does not equal 100%

** Other includes Veterans Affairs medical centers, pediatric emergency departments, and religiously affiliated hospitals.

**Table 2:** Survey responses from emergency physicians in restrictive and semi-restrictive states who reported delays in care of patients with known or suspected ectopic pregnancy (N=24).

Higher threshold of certainty required for ectopic diagnosis	58% (95% CI 37–77%)
Unsure of legality of standard clinical care in my state	29% (14–52%)
Certainty that standard clinical care is legally or institutionally prohibited	25% (11–47%)
Higher threshold of threat to mother’s life	25% (11–47%)
System or peers would not support indicated clinical care	17% (6–39%)

*CI*, confidence interval.

**Table 3:** Survey responses from emergency physicians in restrictive and semi-restrictive states (N=100) about any adaptations of care to stay within legal parameters.

Arranging close follow-up in cases where you might previously have delivered definitive care	31% (95% CI 23–41%)
Additional imaging prior to treatment	26% (18–36%)
Additional B-hcg measurements prior to treatment	24% (17–33%)
Waiting until the patient is more advanced clinically (eg, more pain, hemodynamic instability) prior to treatment	3% (1–9%)
Transfer to a facility with different abortion-related laws	2% (0.5–8%)

*CI*, confidence interval; *B-hcg*, beta-human chorionic gonadotropin.
