# Supplementary material for: Impact of Dobbs on Evaluation and Treatment of Ectopic Pregnancy: National Survey of Emergency Physicians
Source: West J Emerg Med. 2025 Jul 13;26(4):1021–4. doi: 10.5811/westjem.41205 (PMC12342422; doi:10.5811/westjem.41205)
Supplement: Supplementary file 1 [file wjem-26-1021-s001.docx]

Appendix: Survey Questions

Thank you for agreeing to participate in this study.

The following questions relate to your current clinical practice in the emergency department. If you have more than one primary place of practice, please select the one that you consider to be your primary place of practice, and answer all following questions with regard to that primary practice

Since the 2022 Dobbs vs Jackson Supreme Court decision, there have been changes in abortion restrictions and care in many states. We are interested in your experiences practicing emergency medicine in this period of interest (ie, between June 2022 and today).

An ectopic pregnancy is a pregnancy that is outside of the uterus. For purposes of this study, a known/established ectopic pregnancy is one that is radiographically established to be outside of the uterus, and a suspected ectopic is one with positive beta-hcg levels but an ultrasound that shows neither a uterine nor an intrauterine pregnancy.

Demographic Questions (in addition to demographics obtained by InCrowd):

1. Which of the following describe your primary practice (check all that apply):

__ Academic

__Community

__Academic affiliated community

__Religiously affiliated

__Veterans affairs medical center

__Children’s hospital

__Free standing emergency department

1. Which of the following best describes the location of your primary practice?

__Critical access

__Rural

__Suburban

__Urban

1. What is your gender?

__ Female

__ Male

__ Non-binary/non-conforming

__ Transgender

__ Prefer not to reply

Restrictive/Semi-restrictive States:

1. Have you experienced delays in providing clinical care for patients with known or suspected ectopic pregnancy?

If yes:

1a. What is the reason for these delays? (select all that apply)

__Unsure of legality of standard clinical care in my state

__ Certainty that standard clinical care is legally or institutionally prohibited

__ System or peers would not support standard clinical care

__ Higher threshold of certainty required for ectopic diagnosis (ie, more testing or observation needed before treatment compared to before the period of interest)

__ Higher threshold of threat to mother’s life (ie, clinical deterioration or hemodynamic instability) compared to before the period of interest

__ Other

1. Have you adapted your care of patients with known or suspected ectopic pregnancy in any of the following ways to stay within legal parameters compared to before the period of interest? (Select all that apply)

__Arranging close follow up in cases where you might previously have delivered definitive care

__Additional B-hcg measurements prior to treatment

__Additional imaging prior to treatment

__Waiting until the patient is more advanced clinically (e.g., more pain, hemodynamic instability) prior to treatment

__Transfer to a facility with different abortion-related laws

1. During the period of interest, have you discharged patients with established or suspected ectopic pregnancy and told them to seek treatment in a different state?

__No

__Yes

__Unsure/Don’t know

1. During the period of interest, have you transferred patients with established or suspected ectopic pregnancy to a different state for definitive treatment?

__No

__Yes

__Unsure/Don’t know

Permissive/supportive states:

1. In the period of interest, have you noticed an increase in patients from abortion-restricted states coming to your ED for pregnancy-related care?

__No

__Yes

__Unsure / Don’t know

1. In the period of interest, have you received calls from health care providers from other states regarding patients coming to your facility (whether as transfers or recommended presentations) to receive pregnancy care due to restrictions on care in their states?

__No

__Yes

__Unsure / Don’t know

All states:

1. How are you managing a hemodynamically stable patient who has a serum b-hcg of 4000 mIU/mL, abdominal pain, and an empty uterus on pelvic ultrasound?

__Consult ob/gyn for ectopic pregnancy

__Transfer patient for treatment of ectopic pregnancy

__ Arrange repeat b-hcg (+/- repeat ultrasound) in 48 hours to look for abnormal doubling

__ Depends on patient likelihood of reliable follow up

__ Other

1. How are you managing a hemodynamically stable patient who has a serum b-hcg of 500 mIU/mL, abdominal pain, and an empty uterus on pelvic ultrasound?

__Consult ob/gyn for ectopic pregnancy

__Transfer patient for treatment of ectopic pregnancy

__ Arrange repeat b-hcg (+/- repeat ultrasound) in 48 hours to look for abnormal doubling

__ Depends on patient likelihood of reliable follow up

__ Other

1. In the period of interest, has your institution initiated new clinical protocols or care plans for patients with established or suspected ectopic pregnancies that present to your emergency department?

__No

__Yes

__Unsure / Don’t know

**Tables**

| **Table 1:** Survey participant characteristics (N=150) | |
| --- | --- |
| Years since residency graduation [mean (median)] | 13 (12) |
| Gender  [n (%)]    Female    Male    Non-binary | 63 (42%)  86 (57%)  1 (1%) |
| Race  [n (%)]     Asian     Black/African American     White     Other | 22 (15%)  3 (2%)  115 (80%)  3 (2%) |
| Hispanic/Latino  [n (%)] | 5 (3%) |
| Facility location  [n (%)]     Critical access     Rural     Suburban     Urban | 4 (3%)  17 (11%)  71 (47%)  58 (39%) |
| Facility type* [n (%)]     Academic     Academic- affiliated     Community     Free-standing     Other** | 10 (15%)  28 (19%)  70 (47%)  21 (14%)  16 (11%) |
| *Due to rounding, total does not equal 100%  **Other includes VA medical center, pediatric emergency departments, and religiously affiliated hospitals | |

| **Table 2**: Survey responses from emergency physicians in restrictive and semi-restrictive states who reported delays in care of patients with known or suspected ectopic pregnancy (N=24) | |
| --- | --- |
| Higher threshold of certainty required for ectopic diagnosis | 58% (95% C37-77%) |
| Unsure of legality of standard clinical care in my state | 29% (95% CI 14-52%) |
| Certainty that standard clinical care is legally or institutionally prohibited | 25% (95% CI 11-47%) |
| Higher threshold of threat to mother’s life | 25% (95% CI 11-47%) |
| System or peers would not support indicated clinical care | 17% (95% CI 6-39%) |

| **Table 3:** Survey responses from emergency physicians in restrictive and semi-restrictive states (N=100) about any adaptations of care to stay within legal parameters |
| --- |

| Arranging close follow up in cases where you might previously have delivered definitive care | 31% (95% CI 23-41%) |
| --- | --- |
| Additional imaging prior to treatment | 26% (95% CI 18-36%) |
| Additional B-hcg measurements prior to treatment | 24% (95% CI 17-33%) |
| Waiting until the patient is more advanced clinically (e.g., more pain, hemodynamic instability) prior to treatment | 3% (95% CI 1-9%) |
| Transfer to a facility with different abortion-related laws | 2% (95% CI 0.5-8%) |
